# Multiple Painless Cervical Lymphadenopathies Misleading the Diagnosis of Kikuchi’s Disease

**DOI:** 10.7759/cureus.58151

**Published:** 2024-04-12

**Authors:** Afena Apandi, Wan Nabila Wan Mansor, Zalilah Musa, Nurul Atiah Mohd Ali

**Affiliations:** 1 Department of Otorhinolaryngology, Head & Neck Surgery, Faculty of Medicine, Universiti Kebangsaan Malaysia, Kuala Lumpur, MYS; 2 Department of Otorhinolaryngology, Head & Neck Surgery, Hospital Canselor Tuanku Muhriz, Kuala Lumpur, MYS; 3 Department of Otorhinolaryngology, Hospital Sultanah Nur Zahirah, Kuala Terengganu, MYS; 4 Department of Pathology, Hospital Sultanah Nur Zahirah, Kuala Terengganu, MYS

**Keywords:** leukopenia, neck, fever, lymphadenopathy, lymph node, biopsy, histiocyte necrotizing lymphadenitis, kikuchi’s disease

## Abstract

Kikuchi’s disease is an unusual and self-limited disease. It manifests as a painful cervical lymphadenopathy and is associated with a low-grade fever and night sweats. Recently, this disease has been reported worldwide, compared to its initial high prevalence among the Japanese population. The etiologies of Kikuchi’s disease are still unknown, but it has been proposed to have either infectious or immunological causes. We report the atypical presentation of a young male with Kikuchi's disease. A 22-year-old male presented with a prolonged fever for a week, which was associated with bilateral neck swelling that was painless and gradually increased in size. In our case, histopathological examination of the left cervical lymph node revealed histiocytic necrotizing lymphadenitis in favor of Kikuchi's disease. This case report will highlight the atypical clinical presentation of this patient, thereby increasing awareness of the disease's future manifestation.

## Introduction

Kikuchi’s disease is an atypical noncancerous disease that is self-limiting; the presentations may be misdiagnosed with a hematological malignancy such as lymphoma. This disease is common among young adults under the age of 30, and recent reports show that both genders are affected almost equally [1]. It has a higher prevalence among Asian people compared to other populations [2]. The typical presentation is a low-grade fever with painful neck swelling among young patients. It may be associated with a T-cell-mediated immune response in genetically susceptible individuals to a variety of non-specific stimuli [3]. Kikuchi’s disease has no specific hematological blood test or imaging except a diagnostic biopsy. It can be diagnosed by performing fine needle aspiration cytology (FNAC), provided an adequate sample is obtained and performed by a trained cytologist [4]. Since clinical manifestations of the disease may mimic varieties of other life-threatening problems, a few diagnostic modalities are required to gain a diagnosis.

## Case presentation

A 22-year-old male presented with a prolonged fever for one week. He had an intermittent fever, usually in the evening, but it was not associated with chills or rigor. He also had diarrhea and reduced oral intake three days prior to admission. He developed multiple bilateral neck swellings, which were more prominent on the left side for a month. The painless neck swellings increased in size during the fever. There were no systemic symptoms, family history of malignancy, or high-risk behavior.

On neck examination, multiple levels of lymph nodes were palpable: level IV was 2.0 cm x 2.0 cm, level Va was 2.0 cm x 1.0 cm, and the left supraclavicular was 1.0 cm x 1.0 cm. All nodes were non-tender and firm in consistency. Oropharyngeal and otoscopic examinations were normal. Nasal endoscopy and indirect laryngoscopy examinations revealed normal findings. There was no hepatosplenomegaly. The other systemic examination was unremarkable.

A full blood picture revealed leucopenia and thrombocytopenia, with evidence of no leucoerythroblastic picture or blast cells. Infectious disease screening was negative. Inflammatory markers such as C-reactive protein were increased, while the erythrocyte sedimentation rate was normal. Tuberculosis screening, which consists of sputum for acid-fast bacilli and culture, was negative.

**Figure 1:**
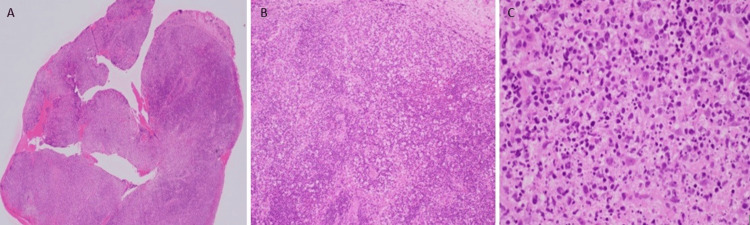
A low-power view at 20 µm magnification 1A: It showed nodal tissue with distorted architecture; 1B: It showed no residual primary follicle; 1C: Collection of eosinophilic, granular material, and karyorrhectic debris with interspersed lymphocytes and histiocytes. It showed no granulomas or atypical cells.

He developed a persistent fever and was treated empirically with intravenous ceftriaxone. He underwent an incisional biopsy under general anesthesia. The histopathological analysis revealed nodal tissue with a distorted architecture (Figure 1). The specimen showed the absence of a primary follicle. There was a collection of eosinophilic, granular, and karyorrhectic debris with interspersed lymphocytes and histiocytes in the absence of granulomas or atypical cells.

**Figure 2:**
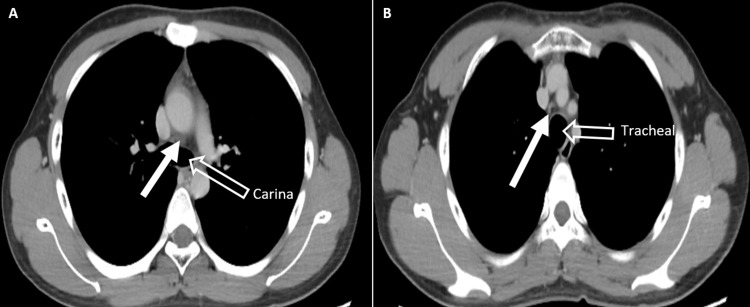
Contrast-enhanced axial CT of the thorax Contrast-enhanced images of the thorax demonstrate non-necrotizing lymph nodes (white arrow) seen in the (A) subcarinal and (B) paratracheal.

A contrast-enhanced computed tomographic scan of the thorax, abdomen, and pelvis showed no evidence of significant intrathoracic or intraabdominal lymphadenopathy. There were a few small, non-necrotizing lymph nodes seen in the subcarinal and paratracheal regions (Figure 2). The largest was in the carina region, measuring 0.6 cm in short axis diameter. Subsequent follow-up revealed that the patient was well, and the neck wound was well healed. He was counseled regarding the risk of recurrence and required surveillance follow-up. He was under surveillance and followed up every six months with a full blood count and lactate dehydrogenase monitoring. 

## Discussion

Kikuchi’s disease, also known as Kikuchi-Fujimoto disease or histiocytic necrotizing lymphadenitis [5], was first reported in 1972 by a group of Japanese people. It was further described as lymphadenitis with focal proliferation of reticular cells, numerous histiocytes, and extensive nuclear debris [6].

Unilateral cervical lymph nodes are more common than bilateral and are usually affected by the posterior triangle cervical node [1]. It was reported that 93% of affected lymph nodes had ranges of 0.5 cm to 4.0 cm. Moreover, 59% were reported to have painful lymphadenopathy [3]. In our case, there were multiple painless cervical lymph nodes in the bilateral neck, and the largest was 2.0 cm x 2.0 cm. It was uncommon compared to the previous reports. Systemic manifestation is usually associated with extranodal involvement; however, it is infrequent in the mediastinal, peritoneal, and retroperitoneal regions.

Imaging techniques such as ultrasonography and computed tomographic examinations do not have specific features of Kikuchi’s disease nodes. However, the purpose of imaging is to look for local extension and systemic involvement of other lymph nodes, such as the mediastinal, peritoneal, and retroperitoneal regions. Other than lymphadenopathy, Kikuchi disease may be presented with a low-grade fever in 50% of cases. It is also associated with a prolonged fever of unknown origin [7]. Thus, clinical suspicion rose when our patient presented with a prolonged fever and painless cervical lymphadenopathy, leading to a biopsy being performed. Systemic symptoms usually manifest when extranodal involvement is present. In this case, the patient presented with a short history of diarrhea, which resolved after five days. Leukopenia was observed in more than 50% of patients, and our case was similar to the one reviewed. There were several autoimmune diseases associated with Kikuchi’s disease, such as antiphospholipid syndrome, polymyositis, systemic juvenile idiopathic arthritis, bilateral uveitis, arthritis, cutaneous necrotizing vasculitis, and pulmonary hemorrhage [3].

Histopathological examination of the left cervical lymph node in our case disclosed histiocytic necrotizing lymphadenitis in favor of Kikuchi’s disease [4]. It was reported in one study that Kikuchi’s disease had undergone multiple stages, such as beginning as proliferative, then necrotizing, and finally resolving into xanthomatous [3]. Microscopic findings showed nodal tissue with a distorted architecture composed of eosinophilic, granular material, and karyorrhectic debris with interspersed lymphocytes and histiocytes [2]. Residual primary follicles are present, but neutrophils and plasma cells are rare or absent. No atypical cells or granuloma formation was seen. Those features favor Kikuchi’s disease diagnosis and exclude tubercular lymphadenitis and lymphoma [8]. Immunohistochemistry was revealed. CD 3 and CD 20 also highlight reactive T and B cells, respectively. Histologically and immunohistochemically, it can be hard to tell the difference between Kikuchi's disease and systemic lupus erythematosus (SLE) lymphadenopathy in some areas. However, the presence of hematoxylin bodies is the most defining histologic feature of SLE lymphadenopathy.

The causative agents for Kikuchi’s disease are still controversial; however, a few microorganisms are potential contributors, such as the Ebstein-Barr virus and herpesviruses 6 and 8 [9]. A previous study mentioned that oral minocycline treatment may contribute to the sensitivity of one of the causative agents for Kikuchi’s disease [10]. In our case, the patient was treated with third-generation cephalosporin, which is excellent against many gram-negative and most gram-positive microorganisms. The patient was treated empirically with intravenous antibiotics.

Kikuchi disease is a self-limiting disease, but cardiac complications should never be taken for granted, as one patient reported dying due to a complication [11]. Others reported complications in a toddler following a febrile illness, as well as in a transplant recipient [3].

The recurrence rate of Kikuchi’s disease was reported as less than 4% [6]. It was reported that two patients developed systemic lupus erythematosus during the five years of surveillance [1]. Thus, patients require regular follow-up for several years to rule out the development of systemic lupus erythematous. Besides, our patient will continue to receive follow-up even though his lymphadenopathy has completely resolved after a month.

## Conclusions

Multiple painless neck swellings and leukopenia may lead to suspicion of a hematological malignancy. However, the clinical presentation was atypical for Kikuchi’s disease, as it commonly presents with a painful unilateral cervical lymph node.

Acknowledgment of this condition can enlighten us and prevent misdiagnosis and unnecessary investigations in managing future patients. A high index of suspicion of Kikuchi’s disease should be kept in mind for those who have been presented with prolonged fever and painless cervical lymphadenopathy.
